# Choosing informative priors in Bayesian regression models: a simulation study and tutorial using Stan and R

**DOI:** 10.3389/fpsyg.2026.1856582

**Published:** 2026-06-24

**Authors:** Daniel Lüdecke, Anna C. Makowski, Jens Klein, Mattan S. Ben-Shachar, Dominique Makowski

**Affiliations:** 1 Institute of Medical Sociology, University Medical Center Hamburg-Eppendorf, Hamburg, Germany; 2 Independent Researcher, Ramat Gan, Israel; 3 School of Psychology, University of Sussex, Brighton, United Kingdom; 4 Sussex Centre for Consciousness Science, University of Sussex, Brighton, United Kingdom

**Keywords:** Bayesian statistics, informative priors, prior elicitation, simulation study, small sample size

## Abstract

**Background:**

Bayesian regression models provide a robust framework for complex data analysis, which is particularly advantageous in scenarios with small sample sizes, common in psychology or medical research. However, specifying appropriate prior distributions that incorporate existing knowledge to regularize model parameters remains a challenge for many researchers. This can lead to unstable or implausible estimates. This study aims to demonstrate the impact of different prior distributions on regression models and to provide a practical guide for choosing and justifying informative priors to produce more stable and credible results.

**Methods:**

The study involved two parts. First, a simulation study was conducted to systematically assess the sensitivity of Bayesian linear regression models to prior specification. We systematically varied the sample size, prior location, and prior scale to observe their impact on posterior estimates for a known true effect size. Second, a case–control study using real-world patient data (*N* = 526) demonstrated the practical application of choosing informative priors. Bayesian logistic regression models were used to analyze the relationship between severe dementia and fall incidence, comparing results from priors based on existing literature (“believer”), conservative priors (“agnostic”), and priors assuming an opposite effect (“skeptical”).

**Results:**

The simulation study showed that strongly informative priors had a substantial influence on posterior estimates, particularly for smaller sample sizes. As the sample size increased, the influence of the data increased, and the estimates converged toward the true effect. In the case–control study, a standard frequentist logistic regression produced an odds ratio of 8.87 with a very wide and unstable confidence interval (1.66–165.19), likely due to data sparsity. In contrast, a Bayesian model using a moderately informative “believer” prior derived from existing research yielded a more stable and plausible odds ratio of 4.01 with a substantially narrower credible interval (1.99–8.78).

**Conclusion:**

Careful and transparent specification of informative priors is a critical tool in Bayesian analysis, especially when data are sparse. By incorporating justified evidence-based assumptions, researchers can regularize models to prevent implausible outcomes and produce more stable, interpretable, and credible results. This approach enhances the robustness of statistical inference in fields where small sample sizes are a frequent challenge.

## 1 Introduction

Bayesian methods present a robust framework to address more intricate research questions, proving particularly advantageous in scenarios where conventional frequentist approaches encounter inherent difficulties and limitations. These limitations include the inability of models to converge to a stable solution, lower accuracy in noisy data and small samples, and proneness to type-I errors (Etz and Vandekerckhove, 2016; Dienes and Mclatchie, 2018). This is especially relevant in fields such as patient studies or randomized controlled trials (RCTs), often characterized by small sample sizes (Nikolakopoulos, 2016), leading to, for instance, challenges in reliably determining the true efficacy and safety of new interventions.

Bayesian regression models, offer solutions to these common issues, namely robust sampling techniques and particularly the possibility of incorporating prior knowledge (Siddique and Aghabazaz, 2023; Gelman et al., 2014). Modeling intricate data characteristics such as heteroscedasticity, individual differences within multilevel models, and obtaining credible intervals for random effects becomes more straightforward within this framework (Rouder and Lu, 2005). This deserves to be highlighted, because multilevel models with random effects are a powerful and important strategy for many study designs in psychology, medical and public health research. For instance, when analyzing patient data from RCTs, these techniques explicitly account for and quantify the variability observed between individual patients. Indeed, certain models prove practically intractable when such analyses are approached using frequentist methods, where the reliance on frequentist *p*-values—especially when strictly focusing on ‘statistical significance’—becomes increasingly difficult to interpret and prone to misleading conclusions (Gelman et al., 2014; McNeish, 2016).

Yet, Bayesian methods remain simple to interpret through their resulting *posterior distributions.* Posterior distributions are probability distributions that show the plausibility of different parameter values given the data and prior beliefs (Kruschke, 2015). This allows for straightforward summaries like mean or median (“point estimate”), and quantiles (“uncertainty intervals”) and enables intuitive and easy-to-understand probability statements for (almost) any quantity of interest, like strength and direction of treatment effects or differences between treatment and control groups (Gelman et al., 2014; Makowski et al., 2019).

A fundamental aspect of Bayesian analysis is the incorporation of prior knowledge by employing prior distributions, in short *priors*. For many practical purposes, priors regularize parameters, effectively excluding or reducing the influence of parameter values that are considered implausible based on existing knowledge or theoretical considerations (Van Erp et al., 2019). This regularization is particularly useful in complex models because it can prevent the model from exploring parameter space regions that are deemed unlikely, thereby averting convergence or singularity issues frequently encountered in non-Bayesian multilevel models. Additionally, in situations where data is scarce, priors provide a sensible range of plausible values, leading to more precise and stable estimates compared with purely data-driven frequentist approaches (McNeish, 2019; van de Schoot et al., 2021). Hence, a key feature of priors is to implicitly steer the model away from solutions that might fit the current data too closely but are likely to exhibit poor generalization to new, unseen data (Etz and Vandekerckhove, 2016; van de Schoot et al., 2021; Banner et al., 2020). Priors are also fundamental for certain types of Bayesian calculations, such as Bayes factors, which are used to quantify the evidence in favor of one statistical model over another. Proper specification of prior distributions is often a prerequisite for Bayes factors to be well-defined and interpretable, because the Bayes factor essentially measures the shift in belief about models from the prior to the posterior (Heck et al., 2023).

However, despite their importance, the task of specifying appropriate priors remains a challenge for many researchers (van de Schoot et al., 2021). This challenge can arise from a variety of factors, including a lack of familiarity with the large number of different prior distributions available and a lack of clear, universally applicable guidelines on how to select them effectively. The practical implementation of Bayesian methods is often perceived as complex, particularly concerning the seemingly subjective nature of prior selection (Banner et al., 2020; Stefan et al., 2022). Addressing this concern by transparent prior selection procedures and comprehensive sensitivity analyses, which allow for the assessment of the impact of different prior choices on the results, is crucial for gaining wider acceptance and adoption of Bayesian methods within the scientific community.

Thus, the present work aims to address two common issues encountered in Bayesian modeling. First, an illustrative simulation study will be carried out to systematically compare the impact and behavior of different types of prior distributions on the posterior distribution of regression models. This step demonstrates how various prior specifications can influence the resulting inferences under controlled conditions. Second, utilizing real-world data from a case–control study, a practical tutorial will be presented to guide researchers through the process of choosing informative prior distributions. This tutorial will not only outline strategies for eliciting and specifying priors based on existing knowledge but will also demonstrate how these choices affect the results obtained from Bayesian regression models. This paper will conclude with concrete recommendations for defining prior information in statistical modeling.

## 2 Materials and methods

We conducted two studies to investigate the impact of priors on Bayesian regression models. First, a simulation study (Study 1) introduces the core concepts of prior definition and demonstrates the relationship between prior influence and sample size. Second, a practical tutorial (Study 2) uses real-world data from a case–control study to illustrate, step-by-step, how to make informed choices for prior elicitation. This tutorial component includes defining priors based on literature and performing a sensitivity analysis to test for potential misspecification or bias. For readers new to Bayesian statistics, the Supplementary file 1 offers a helpful overview of commonly used terms and their meanings.

Due to the different natures of the two studies presented in this paper, the methods are described separately for each study. All computations were based on Stan, a probabilistic programming language for specifying Bayesian models, using Markov Chain Monte Carlo sampling (in particular, Hamiltonian Monte Carlo; Gelman et al., 2014; Carpenter et al., 2017) and its implementation in the statistical programming language R. Hence, all simulations and analyses for this paper were conducted in R, using the packages rstanarm (Bayesian modeling), bayestestR (simulation, posterior summaries), modelbased (prior predictive checks) and ggplot2 (visualization; Makowski et al., 2019; Goodrich et al., 2024; R Core Team, 2025; Wickham, 2016; Makowski et al., 2025). Model diagnostics, such as MCMC convergence, for all Bayesian models were assessed using the bayestestR and performance packages (Makowski et al., 2019; Lüdecke et al., 2021). No issues were detected (e.g., all $\hat{R}$ values were < 1.01 and effective sample sizes were sufficient (for details on MCMC diagnostics, see Gelman et al., 2014; Vehtari et al., 2021). All code and data to reproduce the analyses are available from the osf-repository (Lüdecke and Makowski, 2025).

### 2.1 Study 1: simulation study

We designed a simulation study to systematically assess the sensitivity of Bayesian linear regression models related to the choice of prior distributions. The central aim was to observe how different priors would alter the resulting posterior distribution, which represents our updated beliefs after incorporating the data.

#### 2.1.1 Data simulation

For this analysis, we used basic linear regression models with one predictor and one outcome variable. To ensure a controlled experiment, we simulated the data for each simulation run (see below) with a predefined true effect size. Specifically, we set the underlying correlation between the two variables to 0.3. This process, which included the addition of random noise to prevent a deterministic relationship and data standardization, was carried out using the simulate_correlation() function available in the bayestestR R package (Makowski et al., 2019). This allowed us to generate datasets where the true association was known (a standardized effect size of 0.3), providing a benchmark to evaluate the performance of the models under different prior distributions.

#### 2.1.2 Prior specification

In Bayesian analysis, we formally express our initial beliefs about model parameters through a process called prior specification. This involves selecting a probability distribution where the densest regions correspond to the parameter values we believe are most plausible. Prior distributions are categorized by their level of influence on the outcome. An “informative prior” systematically incorporates established evidence (centered around a specific location parameter), effectively shrinking implausible estimates. A “weakly informative” prior has a mild influence and applies only gentle regularization. Finally, a “non-informative”‘(or “flat”) prior is designed to exert no influence on the final parameter estimates. This yields results comparable to classical frequentist estimates, which can be useful for sensitivity analyses (Robert, 2007). Unlike methods that yield a single point estimate, this approach allows for flexibility in defining the shape and spread of our uncertainty, using distributions like the normal, Student’s t, Cauchy, or Gamma, and many more (Goodrich et al., 2024). For this simulation, we used a normal distribution, which is defined by its mean and standard deviation, or more generally, by its location and scale. In this context, the mean (location) acts as our “best guess” for the parameter’s value based on prior knowledge. The standard deviation (scale), in turn, quantifies our confidence in that guess—a wider distribution signals more uncertainty, while a narrow one signifies a stronger initial belief (Muehlemann et al., 2023). Hence, the prior distribution directly affects the posterior distribution.

**Figure 1:**
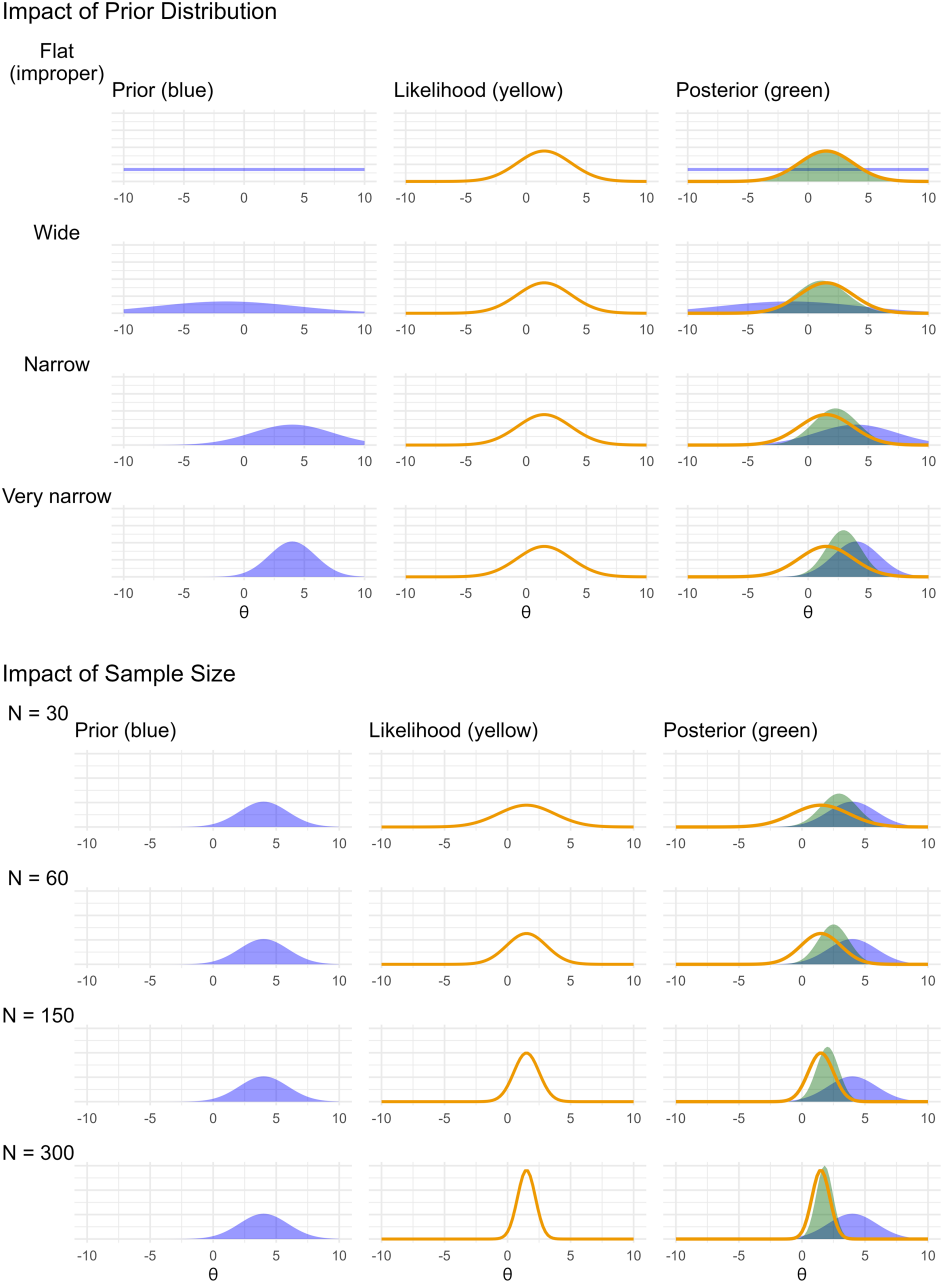
Schematic illustration of the impact of prior shape and sample size on the resulting posterior. The upper panel demonstrates the impact of prior shape (blue) on the posterior distribution (green) when the likelihood (yellow) is fixed, showing how narrower prior scales exert a stronger regularizing pull on the posterior. The lower panel illustrates the impact of increasing sample size (from *N* = 30 to *N* = 300) against a fixed prior. As more data is collected, the likelihood narrows and increasingly dominates the resulting posterior.

To illustrate these mechanics, Figure 1 provides a schematic overview of how the prior distribution and the data (represented by the likelihood) interact to form the posterior distribution. The left panel demonstrates how different prior scales (from a flat to a very narrow prior) increasingly shrink the posterior toward the prior location. The right panel illustrates that, as sample size increases, the likelihood increasingly affects the resulting posterior distribution.

As we simulated correlated data with a true effect size of 0.3 including some variation, we do not expect regression coefficients to be outside of the range of −1 and 1. Therefore, we used four common prior scales that are feasible for standardized effects sizes that are bound to a certain range, the ultranarrow, narrow, medium and wide scale, which correspond to the values $\frac{1}{\surd 27}$ , $\frac{1}{3}$ , $\frac{1}{\surd 3}$ and 1, respectively (Morey and Rouder, 2011). The prior distributions for the intercept and the residual variance were held constant across all simulated models, i.e., weakly informative default priors were assigned to these auxiliary parameters, to isolate our experimental condition.

#### 2.1.3 Simulated scenarios under analysis

We systematically varied sample size, prior location and prior scale to see how different prior specifications behave depending on the available data. Sample size varied from 20 to 200, in increments of 10. Prior locations were −0.6, −0.3, 0, 0.3 and 0.6. Thus, in three scenarios (−0.6, −0.3, and 0), the location (our “guess” about the true effect) was lower than the actual effect of 0.3, and in one scenario, the location was higher (0.6). In total, we had 380 different scenarios (19 different sample sizes * 5 different prior locations * 4 different prior scales). For each scenario, we ran 100 Bayesian linear regression models with one continuous predictor, each time with a newly simulated data set with a true effect size for the predictor of 0.3. This resulted in 38,000 posterior distributions, from which we extracted the median as summary for the point estimate.

#### 2.1.4 Summary of the regression model specification and prior distributions

The notation of the regression model and the Bayesian likelihood is:

$$y_{i}=\beta_{0}+\beta_{1}x_{i}+\varepsilon_{i}$$

$$y_{i}\sim N(\beta_{0}+\beta_{1}x_{i},\sigma^{2})$$

The generic notation of the normal-prior is β~N(μ,σ) , where *μ* (mu) refers to the prior location and *σ* (sigma) to the prior scale. For the simulated linear regression models, the priors for the continuous predictor were systematically varied. The priors were specified as follows for the wide, medium, narrow, and ultranarrow scales:

a) Predictor of Interest (“severe dementia”): Let *β* be the coefficient for the continuous predictor. The scale parameters (*σ*) were defined using the sequence

- Location *μ* = −0.6: β~N(−0.6,1) , β~N(−0.6,13) , β~N(−0.6,13) , and β~N(−0.6,127) .
- Location *μ* = −0.3: β~N(−0.3,1) , β~N(−0.3,13) , β~N(−0.3,13) , and β~N(−0.3,127) .
- Location *μ* = 0: β~N(0,1) , β~N(0,13) , β~N(0,13) , and β~N(0,127) .
- Location μ = 0.3: β~N(0.3,1) , β~N(0.3,13) , β~N(0.3,13) , and β~N(0.3,127) .
- Location μ = 0.6: β~N(0.6,1) , β~N(0.6,13) , β~N(0.6,13) , and β~N(0.6,127) .

b) All other predictors in the model were assigned the standard, weakly informative default prior from the rstanarm package.

### 2.2 Study 2: case–control study

Next, we applied the principles of prior selection to a case–control study, demonstrating how informative priors can be incorporated based on existing literature and expert knowledge. We implemented Bayesian logistic regression models with varying prior specifications and compared the resulting posterior distributions. As additional reference, we also calculated a classical (non-Bayesian) logistic regression model. The transition from a linear model in the simulation to a logistic regression model in the case–control study is intentional, as it allows us to illustrate how the concept of priors applies to a commonly used, but slightly more complex type of regression model in psychology, medical and public health research.

#### 2.2.1 Study design

Data for the second study came from a non-randomized, case–control study carried out in two internal medicine wards in two hospitals in Hamburg, Germany. The original project aimed to compare care quality for dementia patients under a specialized concept versus regular care (Lüdecke et al., 2019). For our current analysis, however, we will focus on the relationship between fall incidence and the severity of cognitive impairments (dementia), using a total sample of 526 patients to demonstrate the use of informative priors. Although an overall sample size of 526 might seem large enough to preclude the need for informative priors, the simultaneous occurrence of severe cognitive impairment and a fall event is rare in this dataset. This sparsity creates issues of (near) complete separation, a scenario in which classical frequentist estimates become highly unstable. Therefore, despite the large total sample, this dataset provides an ideal real-world example to demonstrate the stabilizing effect of priors.

#### 2.2.2 Measures

We incorporated a realistic set of previously established predictors from this study’s earlier work (Lüdecke et al., 2019; Lüdecke and Kofahl, 2020). These encompassed age, gender, length of hospital stay, Charlson Comorbidity Index (CCI; Charlson et al., 2008), and patients’ functional and cognitive status. Functional limitations were measured using the Barthel Index (Mahoney and Barthel, 1965). Our central predictor for the current analysis is the dementia status, which we assessed using the Mini-Mental State Examination (MMSE; Folstein et al., 1975). This tool quantifies cognitive impairment, with scores ranging from zero (indicating very severe impairment) to 30 (very mild or no impairment). We then categorized these scores into three groups: severe dementia (0–16 points), moderate dementia (17–23 points), and mild or no dementia (24–27 points). In our logistic regression model, the categorical dementia predictor is entered using standard dummy coding, with ‘mild or no dementia’ serving as the reference category. The remaining levels (‘moderate’ and ‘severe’) are compared against this reference. We used a binary outcome representing whether the patient experienced a fall or not, which was also recorded during data collection.

#### 2.2.3 Prior specification

To systematically assess the sensitivity of our results to prior information, we treated this as a single regression problem evaluated under varying prior specifications. The underlying model, including the data, covariates, and the Bernoulli likelihood function with a logit link, remained the same. The only components that were altered were the specific prior distributions assigned to the intercept and the severe dementia parameter, based on existing knowledge. It is important to note that there is no universally established terminology for naming prior specifications (Zampieri et al., 2021; Edeh et al., 2025). We aim to avoid imposing bias onto the inference through imprecise or loaded terminology. Hence, the labels employed here are intended to describe the location of the prior distribution relative to the expected effect size and should not be misinterpreted as reflecting a subjective attitude. To clearly distinguish the distinct nature of our prior assumptions, we chose the terms “believer,” “agnostic,” and “skeptical” to describe three different prior locations. The *believer prior location* reflects the expected effect (i.e., the location is chosen close to what our prior beliefs about the parameters are). The *agnostic prior location* is set to zero and reflects a conservative choice. This is appropriate if we have limited or uncertain prior knowledge about an effect, or do not want to introduce a directional bias. Finally, the *skeptical prior location* is set to the opposite of the expected effect, thus being the negative of the believer prior location. It can be used to test and assess the robustness of the findings (e.g., showing that an effect “still holds” despite priors being set “against it”). We then combined the three different locations with three different scales each, using a wide, a narrow and an ultranarrow scale (reflecting a range from minimally to maximally informative). This resulted in nine regression models. Additionally, all models included a prior for the baseline probability of a fall (“intercept”). For all remaining covariates that are not in the focus of this tutorial, we retained the default prior specifications from the rstanarm package (a weakly informative prior).

##### 2.2.3.1 Finding appropriate location parameters for prior distributions

The *believer* prior location is based on prior knowledge and what is usually called *informative prior*. We refer to former research that suggests that dementia increases the risk of falling by two to three times (Kröpelin et al., 2013; van Doorn et al., 2003), depending on the severity of the cognitive impairments. Other research found odds ratios of 1.89 and 3.89 for the association of fall risk and dementia (Monachan et al., 2020; Petersen et al., 2018). For this example, we *simplified* this to an odds ratio (OR) of 3.5 for the link between severe dementia and the risk of a fall (in comparison to the reference category, patients with mild or no dementia). However, in general, the location parameters of priors must be specified on the scale of the linear predictor (as log-odds), not as OR. Therefore, we converted our assumption to its log-odds value, which is approximately 1.25 for severe dementia ( $e^{1.25}\sim 3.5$ ). The same logic of transformation would also apply for other generalized regression models, like Poisson regression. The *agnostic* prior location is in general set to zero, thereby not requiring any further prior knowledge. The skeptical prior is set exactly to the negative of the believer prior, which is −1.25. This specific value is not arbitrary. Rather, it indicates the strict opposite of the optimistic belief, useful to test the sensitivity of prior choice.

Next, we incorporated existing knowledge about the outcome itself. This is accomplished by placing a prior on the model’s intercept. Because we are using dummy coding for ‘dementia’, the model’s intercept represents the baseline probability of a fall for our reference category, patients with mild or no dementia. While research shows that fall rates in dementia patients can vary widely (from 10% up to 40%), some studies suggest a narrower probability of 10–15% (Tängman et al., 2010; Davey et al., 2024). Based on this, we assumed that the probability of falling is higher for patients with severe dementia than for those with mild or no dementia (the reference category, represented by the intercept). For this example, again simplifying, we therefore chose a 5% probability of fall for our reference category. Again, since logistic regression models work on the log-odds scale, we had to convert this percentage. A 5% probability translates to a log-odds value of approximately −2.95, which we then used as the prior location for the intercept (R code to calculate the log-odds: *qlogis(0.05)*).

It is important to remember that Bayesian priors do not treat these location parameters as rigid, frequentist point estimates. Rather, they serve merely as the center of the distribution. The advantage of priors is to take the uncertainty found in the literature into account (e.g., the spread of ORs from 1.89 to 3.89, or the varying fall rates of 10 to 40%), which is handled by the prior’s scale parameter, which is detailed in the following section.

##### 2.2.3.2 Finding appropriate scale parameters for the prior distributions

For severe dementia, we tested three different levels of uncertainty (scale parameters) in our prior specification. The wide (minimally informative) prior scale was set to 2.5, which corresponds to the default scale utilized by the rstanarm package when no prior distributions are specified for a parameter. This value is derived from established recommendations for weakly informative priors in logistic regression, which assigns low probabilities to unrealistically extreme effect sizes but still allows for a wide range of plausible effect sizes (Gelman et al., 2008). To ensure the prior remains appropriately weakly informative regardless of the original metric of the data, the package internally automatically scales this parameter based on the standard deviation of the predictor (e.g., adjusting the scale to 2.5 / (2* SD) for continuous numeric predictors, Gelman et al., 2008). The narrow prior scale utilized a value of 0.5, reflecting a moderate degree of uncertainty. This is the recommended scale for logistic regression models (Ghosh et al., 2018). This value implies that we have a prior belief the true OR for severe dementia ranges between about half ( OR=1.28 , the 5th prior quantile) and more than twice ( OR=9.59 , the 95th prior quantile) the size of our most likely estimate [location parameter, OR = 3.5; R code to calculate the interval: exp(1.25 + 0.5 * qt(c(0.05, 0.95), df = 5))]. The ultranarrow (maximally informative) prior scale is set to 0.2, chosen for illustrative purposes to represent highly confident beliefs. The value of 0.2, for example, creates a very strong prior that assumes the true OR is almost certainly between 2.34 and 5.24, which is a very high degree of certainty.

A scale parameter of 0.5 was chosen for the prior for the intercept, reflecting a reasonable degree of uncertainty (Ghosh et al., 2018). This allows the probability of a fall (for the reference group, mild or no dementia) to plausibly range from approximately 2 to 13% [R code to calculate these probabilities: plogis(qlogis(0.05) + 0.5 * qt(c(0.05, 0.95), df = 5))], capturing a realistic spectrum of incidence rates across different hospital settings.

##### 2.2.3.3 Finding an appropriate prior distribution

The selection of a prior distribution should be guided by the plausible region of possible parameter values. For instance, while a normal distribution is suitable for regression coefficients that can either be positive or negative, this is inappropriate for parameters that are strictly positive, such as variance components for random effects. In such cases, a distribution that is only defined for positive values, like the Gamma distribution, is a more logical choice. With the parameters in our case–control study, effect sizes can plausibly be negative or positive.

**Figure 2:**
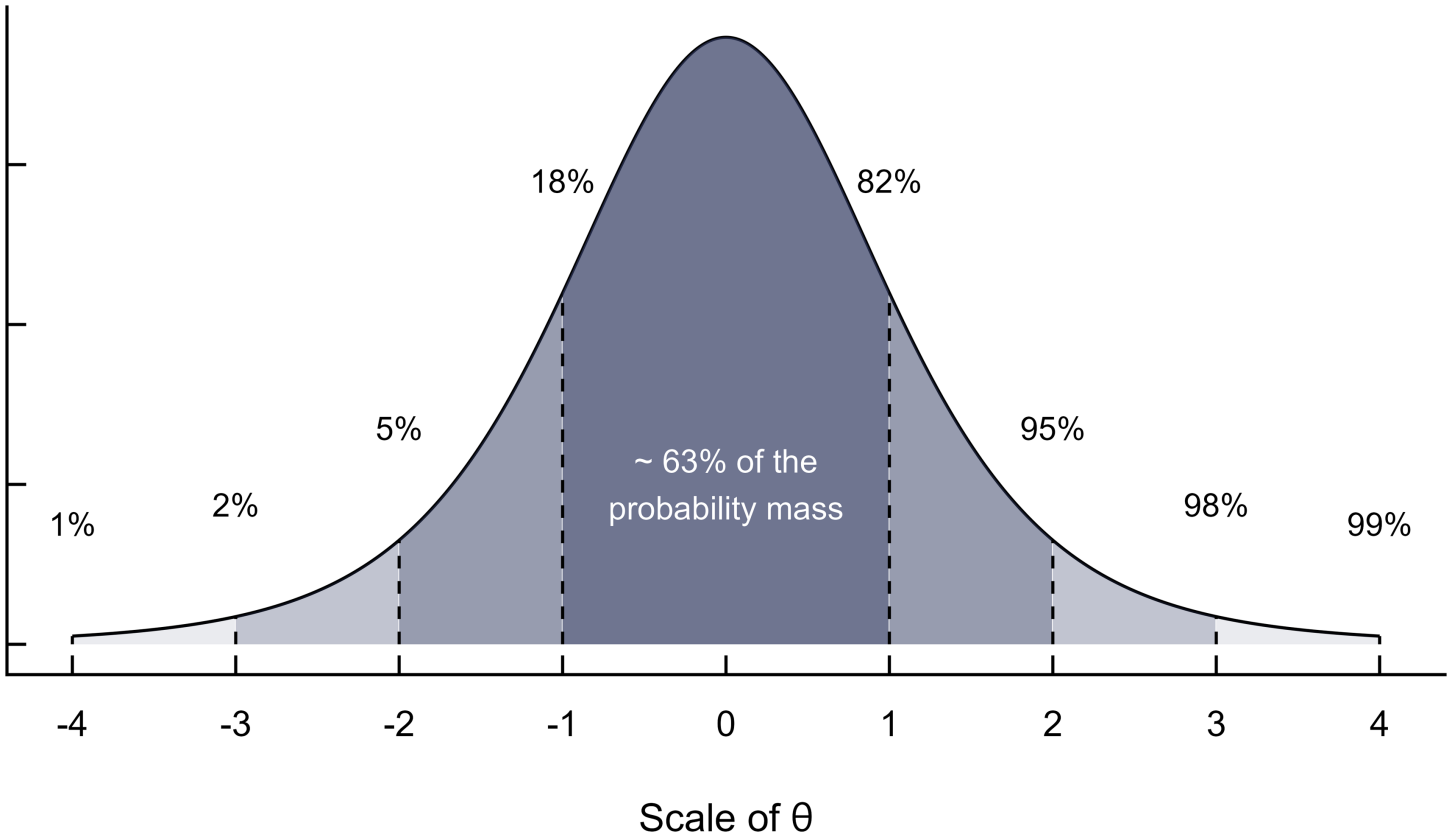
Probability mass for Student’s t-distribution with 5 degrees of freedom. The distribution illustrates the expected spread of plausible parameter values for a standardized scale. Dashed lines indicate the boundaries capturing approximately 63, 82, 95, 98, and 99% of the probability mass, showing how chosen scale parameters constrain the expected effect sizes.

For logistic regression models, studies suggest that a Student’s t-distribution with five degrees of freedom is more appropriate than a normal distribution (Ghosh et al., 2018). For this distribution, we know that 63% of the probability mass lies within +/− 1 scale unit of a distribution (see Figure 2). Thus we can say that—if we use a (narrow) unit scale of 0.5—we believe the most likely outcome is an OR of 3.5 (the location parameter), but we assume there is a high chance (of 63%) that the outcome could range from 2.12 ( $e^{\log(3.5)-0.5}$ ) to 5.77 ( $e^{\log(3.5)+0.5}$ ). Other ORs are also plausible, but less likely (see Figure 2).

##### 2.2.3.4 Summary of regression model specification and prior distributions

The notation of the regression model was specified as follows. Let $Y_{i}$ be the binary outcome indicating whether patient i experienced a fall or not. We assume $Y_{i}$ follows a Bernoulli distribution with probability $P_{i}$ :

$$Y_{i}\sim \text{Bernoulli}(P_{i})$$

The log-odds (logit) of a fall event was modeled as follows, where $\beta_{2}$ indicates the coefficient for “severe dementia” (our predictor of interest):

$$\begin{array}{l} \text{logit}(P_{i})=\ln(\frac{P_{i}}{1-P_{i}})=\beta_{0}+\beta_{1}(\text{moderate dementi}a_{i})+\\ \beta_{2}(\text{severe dementi}a_{i})+\beta_{3}(\mathrm{ag}e_{i})+\beta_{4}(\mathrm{se}x_{i})+\\ \beta_{5}(\text{length of}\;\mathrm{sta}y_{i})+\beta_{6}(\mathrm{CC}I_{i})+\beta_{7}(\text{Barthe}l_{i}) \end{array}$$

The generic notation of the Student’s t-prior with 5 degrees of freedom is θ~t(ν,μ,σ) , where *v* (nu) are the degrees of freedom, *μ* (mu) refers to the prior location and *σ* (sigma) to the prior scale. The priors for the full logistic regression model were specified as follows, for wide, narrow and ultranarrow scales:

a) Intercept (baseline fall risk): Intercept~t(5,−1.9,0.5)
b) Predictor of Interest (“severe dementia”): Let *θ* be the coefficient for severe dementia.

- Believer priors: θ~t(5,1.25,2.5),θ~t(5,1.25,0.5), and θ~t(5,1.25,0.2).
- Agnostic priors: θ~t(5,0,2.5),θ~t(5,0,0.5), and θ~t(5,0,0.2).
- Skeptical priors: θ~t(5,−1.25,2.5), θ~t(5,−1.25,0.5), and θ~t(5,−1.25,0.2).

All other predictors in the model were assigned the standard, weakly informative default prior from the rstanarm package, θ~t(5,0,1).

#### 2.2.4 Validating formal priors with prior predictive checks

Thus far, our prior specifications have been derived from previous research (informing location parameters) and methodological recommendations for logistic regression (informing parameters such as scale and degrees of freedom). However, it is crucial to verify whether these mathematical definitions accurately represent the assumed underlying reality. Domain knowledge suggests that the probability of a fall likely ranges between 5 and 29%, and that patients with severe dementia face higher odds of falling compared to those with mild or no cognitive impairment. These expectations must be consistently reflected in the prior distribution (van de Schoot et al., 2021; Gabry et al., 2019).

One common method to validate our priors is the prior predictive checking, where data is sampled from the model using only the prior distributions (prior predictive distribution), before any observed data is considered. By visualizing the prior predictive distribution, researchers can assess whether their chosen priors generate plausible data, providing a crucial sanity check on the model’s assumptions and their consistency with domain knowledge.

To perform a prior predictive check, one can simply adjust the call to the model fitting function to sample solely from the prior distribution. From there, we predict the probability of a fall event relative to the predictor of interest, dementia severity. The resulting pattern shows whether the chosen prior distribution aligns with our real-world assumptions. For this tutorial, we show the prior predictive checks for the believer, agnostic and skeptical prior using the recommended narrow scale of 0.5, and additionally an inappropriate prior specification [weakly informative prior specified as normal distribution with large variance, N(0,5) ], to illustrate the visual distinction between a well-calibrated prior and a misspecified one.

#### 2.2.5 Statistical inference and decision rules

In alignment with the Bayesian Analysis Reporting Guidelines (Kruschke, 2021), it is necessary to clarify our approach to statistical inference. Because the primary pedagogical goal of this tutorial paper is to illustrate the regularizing impact of prior distributions, we did not adopt a dichotomous decision rule for hypothesis testing (e.g., utilizing Bayes Factors, Regions of Practical Equivalence (ROPE), or Bayesian *p*-values). Instead, our inference relies on continuous estimation. We summarize the posterior distributions using the posterior median as our point estimate for the Odds Ratios (OR) and the 95% equal-tailed credible interval to quantify uncertainty. We maintained the OR scale because it is a familiar measure of effect size for clinical and psychological research. Furthermore, prior predictive checks were employed exclusively to validate the plausibility of the prior space (see Section 3.2.4), rather than serving as an inferential test of the posterior.

## 3 Results

**Figure 3:**
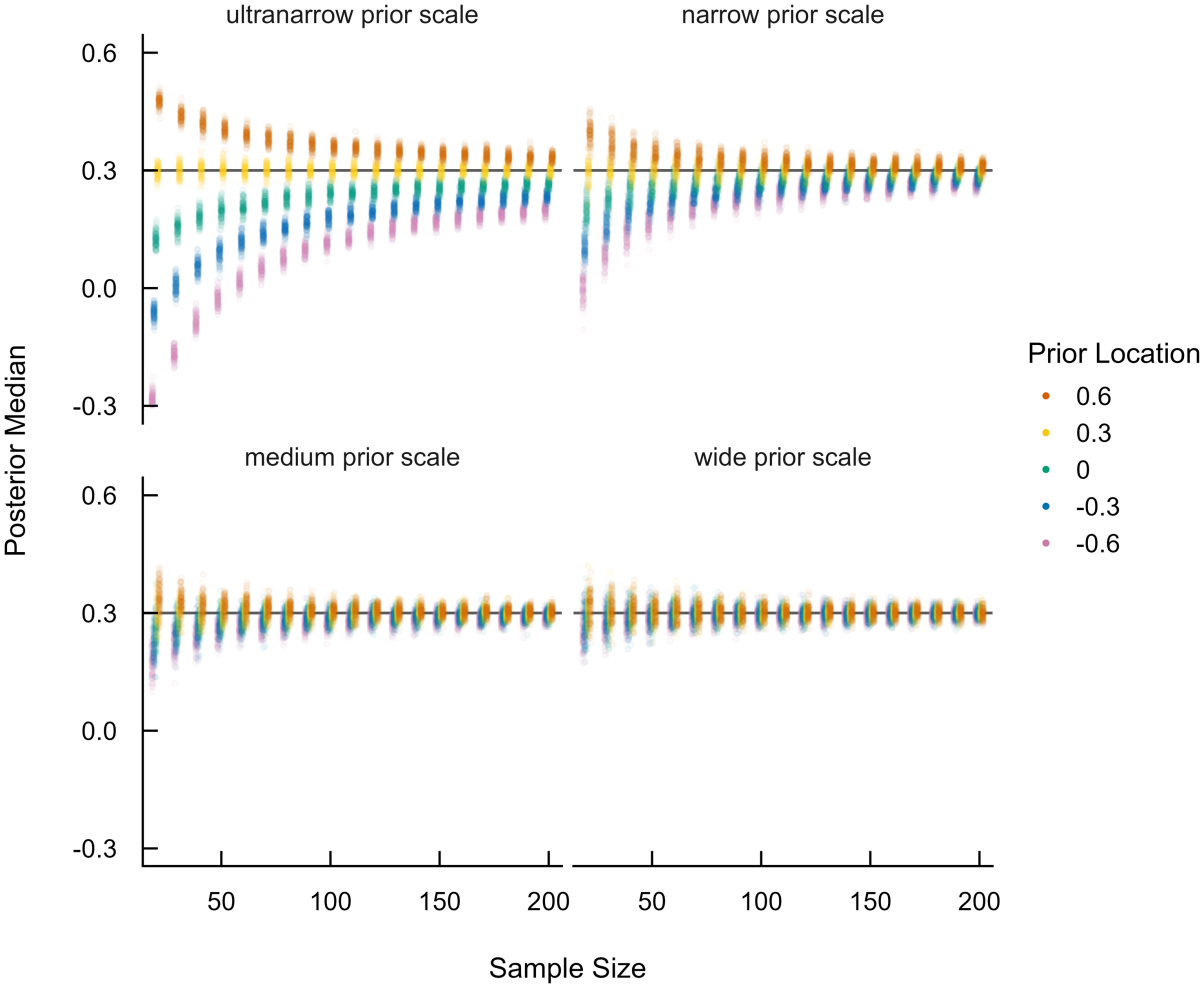
Posterior median by sample size, prior location and prior scale for true effect of 0.3. Results from 100 regression models with simulated data for each combination of sample size, location, and scale. Narrower prior scales strongly pull estimates toward the prior location at smaller sample sizes. As sample size increases, the data dominates the prior, allowing the estimates to converge on the true effect regardless of the initial prior location.

Figure 3 illustrates how the posterior median, or the estimated effect size, is influenced by sample size, prior location (the initial “guess”), and prior scale (the level of certainty). The results show that when priors are very certain (ultranarrow or narrow scale), the model’s estimate is strongly pulled toward the prior’s location, especially for small sample sizes. As the sample size increases, the data gains influence, and the estimates gradually converge toward the true effect of 0.3. Conversely, with less certain priors (medium or wide scale), the data has a much stronger impact, and the estimates are consistently close to the true effect, even with smaller sample sizes, regardless of the initial prior location.

**Figure 4:**
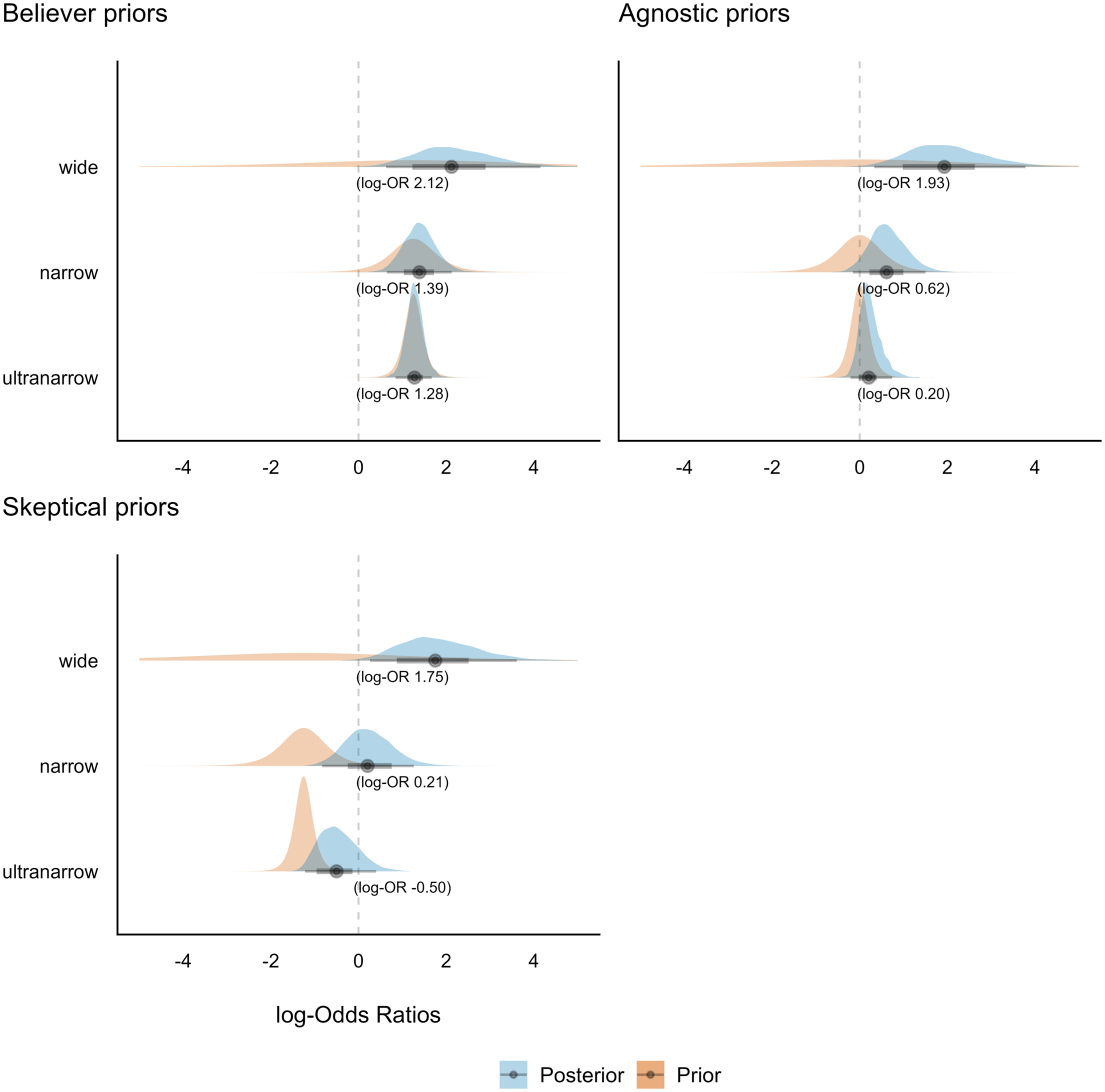
Prior and posterior distributions for the effect of severe dementia on fall risk. Results from the case–control study are shown on the log-odds scale across varying prior locations (believer, agnostic, skeptical) and prior scales (wide, narrow, ultranarrow). As the prior scale becomes narrower, it exerts stronger regularization, pulling the posterior distribution closer to the prior location.

The results from the case–control study demonstrate the impact of prior specification on model outcomes, as detailed in Table 1 and illustrated in Figure 4. The standard frequentist logistic regression produced a very large odds ratio (OR) of 8.87 for the effect of severe dementia on fall risk, but with an extremely wide confidence interval of 1.66–165.19. The Bayesian models show how different prior assumptions systematically influence the results. When using “believer” priors obtained from the existing literature, a wide scale produced an OR of 8.36, close to the unstable frequentist result. As the prior became more informative with a narrow scale, the OR was regularized to a more plausible 4.01 with a substantially tighter credible interval (1.98–69.57). The agnostic prior, which is more conservative, reduced the OR to 6.90 even with a wide scale and shrinking it further to 1.85 (narrow scale) and 1.23 (ultranarrow scale). Using a skeptical prior to assume a protective effect, the wide scale still produced a large positive OR of 5.77. This effect was shrunk to just 1.23 and 0.61 under narrow and ultranarrow skeptical priors, respectively. Figure 4 visually confirms these patterns, showing the posterior distribution (in blue) being pulled from the data’s likelihood toward the specified prior distribution (in orange). The stronger the prior (i.e., the narrower the scale), the more influence it exerts.

**Table 1:** Odds Ratio and equal-tailed 95% confidence/credible intervals for the coefficient “severe dementia,” for frequentist logistic regression model and the nine Bayesian models with different prior information.

Model	OR (severe dementia)	95% CI
Frequentist log. Regression	8.87	1.66–165.19
Believer prior location		
Wide scale	8.36	1.98–69.57
Narrow scale	4.01	1.99–8.78
Ultranarrow scale	3.59	2.42–5.55
Agnostic prior location		
Wide scale	6.90	1.59–53.06
Narrow scale	1.85	0.89–4.78
Ultranarrow scale	1.23	0.85–2.28
Skeptical prior location		
Wide scale	5.77	1.43–42.89
Narrow scale	1.23	0.49–4.08
Ultranarrow scale	0.61	0.31–1.63

**Figure 5:**
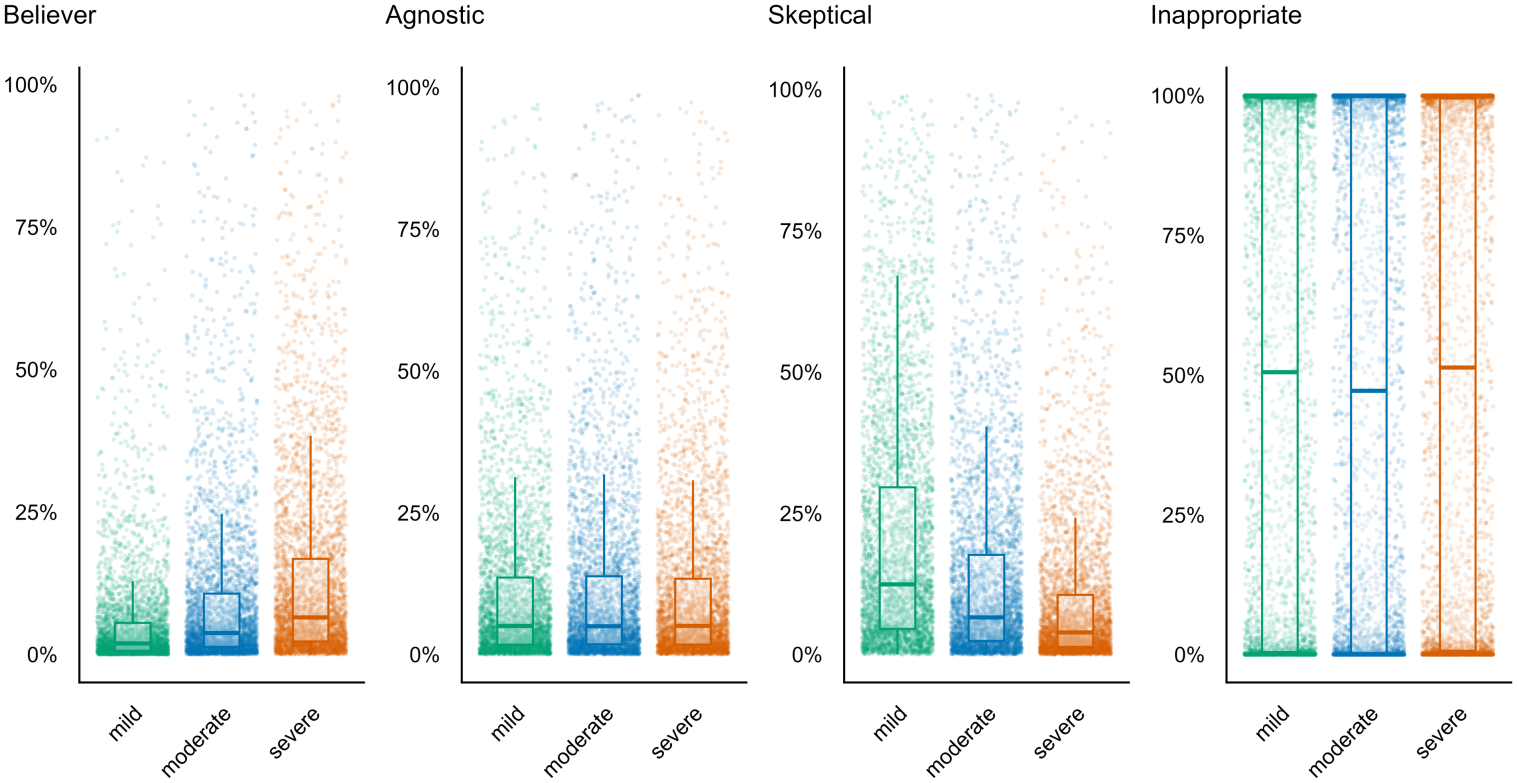
Prior predictive checks for the case–control study. The plots show the simulated prior predictive distributions of fall probabilities across the three dementia severity groups, with overlaid boxplots highlighting the median and interquartile ranges of the probability mass. The rightmost panel demonstrates an inappropriate, poorly calibrated prior that incorrectly pushes the probability mass to the absolute extremes of 0 and 1.

Figure 5 demonstrates results from prior predictive checks. The overlaid boxplots emphasize the distribution of the probability mass of the prior predictive distributions. This visualizes how all three priors allow for a wide range of probabilities, with lower probabilities of falling generally being more likely. The *believer* prior tends to assign higher fall probabilities to patients with severe dementia, the *agnostic* prior gives approximately equal probabilities to all three dementia groups, while the *skeptical* prior gives higher fall probabilities to those with mild or no cognitive impairments. Finally, the inappropriate prior (right panel) demonstrates poor calibration by polarizing the predictions. It pushes probability mass almost exclusively to the extremes of 0 and 1, leaving the plausible middle range largely unsupported.

## 4 Discussion

The aim of this paper was to demonstrate the impact of different prior distributions and to provide a practical guide for choosing them. Through a simulation study and a case–control tutorial, we highlighted how the careful specification of priors—particularly the scale and location parameters—can lead to more stable and credible results. Our findings underline that transparently chosen informative priors are especially useful in scenarios with small sample sizes or sparse data, which are common in psychology, medical and public health research.

The simulation study provided insights into the interplay between prior influence and sample size. The results clearly showed that maximally informative (i.e., narrow scale) priors exert considerable influence on posterior estimates when data is sparse, pulling the results toward the prior location. With a larger sample size, the influence of the prior distribution diminished while the impact of the data increased, and the estimates converged toward the true effect. Conversely, weakly, minimally informative (i.e., wide scale) priors allowed the data to dominate the inference regardless of sample size. This finding is critical as it illustrates a fundamental concept for researchers: the prior acts as a regularizing force and its strength must be weighed against the strength of the available data. For studies with limited sample sizes, a well-justified informative prior can prevent a model from producing highly uncertain or implausible estimates. However, regularization should not be applied naively. Informative priors should be carefully justified to avoid artificially suppressing uncertainty in situations where a genuine lack of knowledge exists.

The case–control study served as a practical tutorial, demonstrating the application of priors in logistic regression, a common choice in psychological, medical or public health research for binary outcomes. The frequentist model yielded an OR with an extremely wide confidence interval, making the result nearly uninterpretable. This happens when data is sparse or when we only have very few observations within one group (in this case: severe dementia; Clark et al., 2023). By incorporating knowledge from the literature into a moderately informative “believer prior” (assuming an OR of 3.5, location = 1.25 with a scale of 0.5), we obtained a plausible OR of 4.01 with a substantially narrower and more precise credible interval. Hence, even with an adequate overall sample size, in cases of (near) complete separation, the use of informative priors offers a robust alternative to often unstable classical maximum likelihood estimation. The tutorial further demonstrated how to formally assess the finding’s robustness by testing it against “agnostic” (location = 0) and even “skeptical” (location = −1.25) priors. Notably, even when the prior assumed a protective effect, the impact of the data was strong enough to produce a positive OR when the scale was wide, underscoring the strength of the observed association. Only the ultranarrow scale for the skeptical prior yielded an OR lower than 1 for the median of the posterior distribution.

This use of priors can be seen as the Bayesian equivalent of frequentist regularization. A well-established frequentist approach is Firth’s logistic regression, which employs a so-called penalized likelihood method that effectively shrinks the coefficients, correcting the bias introduced by separation (Clark et al., 2023; Firth, 1993). The Bayesian approach achieves the same results by specification of priors. Priors inherently act as a form of regularization, preventing model parameters from diverging to infinity and pulling estimates toward more plausible values (Mansournia et al., 2018). This reveals that the use of an informative prior is not purely subjective. Firth’s correction is equivalent to a Bayesian analysis that uses a specific, non-informative prior known as the Jeffreys’ invariant prior (Gelman et al., 2008; Kosmidis and Firth, 2021). Other penalized regression methods also have direct Bayesian interpretations; for example, ridge regression corresponds to placing a normal (Gaussian) prior on the regression coefficients, while LASSO regression is analogous to a Laplace prior (Šinkovec et al., 2021; Tibshirani, 1996). Unlike Firth’s correction or ridge regression, which use very generic priors, the Bayesian framework allows researchers to incorporate more specific, context-dependent information to achieve more accurate and justifiable regularization. This makes the Bayesian approach highly relevant even for researchers primarily trained in frequentist methods.

The simulation study’s core finding—that the influence of the prior diminishes as sample size increases—is a foundational concept in Bayesian statistics. For large sample sizes, the information from the data comes to dominate the prior distribution. As a result, the posterior distribution converges toward the maximum likelihood estimate, becoming robust to the specific choice of the prior. This finding is in line with other simulation studies that consistently show that with smaller samples, posterior results more heavily rely on the prior specification (Van De Schoot et al., 2015; Jones et al., 2022). As our simulation demonstrates, a biased or misspecified prior (e.g., with location −0.6 when the truth is 0.3) has a substantial effect on the posterior when the sample size is very small. Our simulation provides an accessible guide to how priors can be leveraged to provide stability and guard against unreasonable or highly uncertain estimates in such situations (Thirard et al., 2020). Especially for small samples, we recommend conducting a sensitivity analysis by comparing results from different prior specifications (e.g., the believer, agnostic, and skeptical priors from our case study).

Prior elicitation should be accompanied by visual checks, so called prior predictive checks. These serve as a sanity check to validate our formal priors. The believer prior favored higher fall risks for severe dementia, while the skeptical prior favored the reverse. This visualization step revealed that our priors were distinct and operated as intended, validating that the mathematical definitions (location and scale) aligned with our assumptions about the relationship between dementia severity and fall risk that we wanted to test.

A primary concern for researchers is the perceived subjectivity of prior selection. This is often seen as a threat to scientific objectivity, leading many researchers to default to so-called “non-informative” priors to let the data “speak for itself” (Banner et al., 2020; Stefan et al., 2022). However, any statistical analysis, whether Bayesian or frequentist, is built upon assumptions that are “subjective” in the sense that they are chosen by the researcher based on background knowledge. In this regard, Bayesian analysis is no more subjective than frequentist approaches. Our work demonstrates that, particularly within the Bayesian framework, this critique of subjectivity can be mitigated through transparency and explicit justification (Gelman and Hennig, 2017).

But what if no prior research or expert opinion is available? Non-informative (flat, uniform) priors should be avoided in certain situations. For instance, as we have seen in our case–control study, data sparsity or (near-) complete separation can lead to the unstable and misleading estimates produced by maximum likelihood (which is equivalent to a Bayesian model with flat priors; Gelman et al., 2008; Clark et al., 2023). The recommended minimum is a weakly informative (“agnostic”) prior (van de Schoot et al., 2021; Banner et al., 2020; Stan Development Team, 2026). These priors are conservative because they gently “pull” the estimated effect toward zero, decreasing the risk of type I Error (i.e., incorrectly concluding that an effect exists when the true effect is zero). However, the pull is weak because the wide scale only rules out extreme, highly unlikely values, providing a subtle form of regularization (Goodrich et al., 2024; Stan Development Team, 2026). It should be noted, however, that a flat prior is acceptable, for instance, if you know that data sparsity or (near-) complete separation is not a problem. In such cases, results from frequentist and Bayesian models are often very similar (van de Schoot et al., 2021; Jones et al., 2022).

Based on our simulation study and practical tutorial, and formerly recommendation on eliciting priors, we propose the following guidelines for choosing informative priors in statistical modeling (Table 2; van de Schoot et al., 2021; Zondervan-Zwijnenburg et al., 2017; Lemoine, 2019):

**Table 2:** Recommendations for prior elicitation.

Principle	Recommendations
1. Grounding your prior location (the “guess”)	*Believer Prior:* When strong evidence based on literature or expert opinion is available, set the prior location to reflect the expected effect size (e.g., an OR of 3.5, or log-odds of 1.25). This directly incorporates domain knowledge into the model. *Agnostic Prior:* When knowledge is limited or a conservative baseline is needed, set the location to zero. This is a standard approach for creating a *weakly informative* prior that assumes no effect. *Skeptical Prior:* To formally test the robustness of your findings, set the location to the opposite of the expected effect. Should the data overcome this skeptical prior, it provides strong evidence for the effect.
2. Defining uncertainty (the scale)	*Wide Scale:* Use when you are very uncertain about the prior location and want the data to have the most influence. This is a good default for agnostic priors. *Narrow Scale:* Use when you have more confidence in your prior location, based on existing evidence. This provides stronger regularization and leads to estimates that are more precise. *Ultranarrow Scale:* Use with extreme caution, as it makes the prior highly influential. This is only appropriate when the prior information is exceptionally strong and well-justified.
3. Ensuring robustness and transparency	*Conduct sensitivity analysis:* Run models using believer, agnostic, and skeptical priors as a built-in sensitivity analysis. Comparing the results shows how dependent the findings are on the initial assumptions. *Visualize priors and posteriors:* Always plot the prior and posterior distributions to visually assess how much the prior influenced the result, as demonstrated in Figure 4. *Justify and report*: Clearly state the rationale for your choice of prior location and scale, explaining how they were informed by literature or your research goals. Transparency is essential for credibility.
4. Choosing the prior distribution shape	For parameters that can be positive or negative (like regression coefficients), a Student’s t or normal distribution is appropriate. For parameters that must be positive (like random effect variances), use a distribution defined on the positive domain, such as the Gamma distribution
5. Validate prior using visual checks	Conduct prior predictive checks to confirm that the prior distributions map correctly onto our assumptions about the true relationship between outcome and predictor.

### 4.1 Limitations

This study has some limitations. The simulation was constrained to specific models and prior distributions, and its findings may not generalize to all contexts. Similarly, the case–control study represents a single, albeit illustrative, example of prior elicitation. Future research should focus on developing clearer guidelines for prior selection across different types of models and research fields. Investigating data-driven or automated approaches to prior elicitation could also be valuable, especially when expert knowledge is limited. For instance, there are recent developments regarding prior-sensitivity analysis that help detecting misspecified priors or when information from data is scarce (Kallioinen et al., 2024). We showed one such method, the prior predictive checks. However, we only demonstrated a convenient way to carry out these validation steps. There are further, more advanced ways to conduct prior predictive checks that also involve data simulation (van de Schoot et al., 2021; Gabry et al., 2019). Similarly, our tutorial does not cover other formal approaches for creating informative priors, such as the power prior. This approach is common in fields like clinical trials where data from a specific, similar previous study is available. Instead of using a literature review to form a general belief (like in our case study), the power prior directly incorporates the actual data from previous studies (Ibrahim et al., 2015). Finally, providing a comprehensive, step-by-step framework for model selection and formal hypothesis testing falls beyond the scope of this paper. For researchers seeking advanced guidance on moving from sensitivity analyses to final decision-making or formal Bayesian hypothesis testing, we direct readers to more comprehensive textbooks on Bayesian data analysis (Gelman et al., 2014; Kruschke, 2015; McElreath, 2016).

## 5 Conclusion

Bayesian methods offer a powerful and flexible framework for analyzing complex data, particularly in situations with small sample sizes. The ability to incorporate prior knowledge through prior distributions provides significant advantages in terms of regularization, model stability, and interpretability. This study demonstrates the impact of prior specification in Bayesian regression modeling, providing a practical tutorial and simulation study. We recommend that researchers carefully consider the choice of prior distributions, leveraging prior knowledge and assessing the sensitivity of results to prior specification.

Future research could explore the development of more automated or data-driven approaches for prior elicitation, particularly in situations where expert knowledge is scarce. Investigating the performance of different prior selection strategies across a wider range of model types and data structures would also be beneficial. Furthermore, the broader implementation of the principles and proven methods of Bayesian modeling in psychology and medical research should be encouraged. It is important to address concerns about “subjective arbitrariness” by highlighting that scientific studies often include subjective decision-making, with or without a Bayesian approach—highlighting the responsible application of statistical methods.

## Data Availability

The datasets presented in this study can be found in online repositories. The names of the repository/repositories and accession number(s) can be found at: https://doi.org/10.17605/OSF.IO/8EVY5.

## References

[ref1] Banner K. M. Irvine K. M. Rodhouse T. J. (2020). The use of Bayesian priors in ecology: the good, the bad and the not great. Methods Ecol. Evol. 11, 882–889. doi: 10.1111/2041-210X.13407

[ref2] Carpenter B. Gelman A. Hoffman M. D. Lee D. Goodrich B. Betancourt M. . (2017). Stan: a probabilistic programming language. J. Stat. Softw. 76, 1–32. doi: 10.18637/jss.v076.i01, 36568334 PMC9788645

[ref3] Charlson M. E. Charlson R. E. Peterson J. C. Marinopoulos S. S. Briggs W. M. Hollenberg J. P. (2008). The Charlson comorbidity index is adapted to predict costs of chronic disease in primary care patients. J. Clin. Epidemiol. 61, 1234–1240. doi: 10.1016/j.jclinepi.2008.01.006, 18619805

[ref4] Clark R. G. Blanchard W. Hui F. K. C. Tian R. Woods H. (2023). Dealing with complete separation and quasi-complete separation in logistic regression for linguistic data. Res. Methods in Applied Linguistics 2:100044. doi: 10.1016/j.rmal.2023.100044

[ref5] Davey N. Connolly E. Mc Elwaine P. Kennelly S. (2024). A systematic review of falls risk of frail patients with dementia in hospital: Progress, challenges, and recommendations. CIA 19, 1127–1139. doi: 10.2147/cia.s400582, 38948169 PMC11214555

[ref6] Dienes Z. Mclatchie N. (2018). Four reasons to prefer Bayesian analyses over significance testing. Psychon. Bull. Rev. 25, 207–218. doi: 10.3758/s13423-017-1266-z, 28353065 PMC5862925

[ref7] Edeh E. Liang X. Cao C. (2025). Probing beyond: the impact of model size and prior informativeness on Bayesian SEM fit indices. Behav. Res. Methods 57:108. doi: 10.3758/s13428-025-02609-2, 40042526 PMC11882663

[ref8] Etz A. Vandekerckhove J. (2016). A Bayesian perspective on the reproducibility project: psychology. PLoS One 11:e0149794. doi: 10.1371/journal.pone.0149794, 26919473 PMC4769355

[ref9] Firth D. (1993). Bias reduction of maximum likelihood estimates. Biometrika 80, 27–38.

[ref10] Folstein M. F. Folstein S. E. McHugh P. R. (1975). “Mini-mental state”. A practical method for grading the cognitive state of patients for the clinician. J. Psychiatr. Res. 12, 189–198. 1202204 10.1016/0022-3956(75)90026-6

[ref11] Gabry J. Simpson D. Vehtari A. Betancourt M. Gelman A. (2019). Visualization in Bayesian workflow. J. Royal Statistical Society Series A: Statistics in Society 182, 389–402. doi: 10.1111/rssa.12378

[ref12] Gelman A. Carlin J. B. Stern H. S. Dunson D. B. Vehtari A. Rubin D. B. (2014). Bayesian data analysis. Third Edn Boca Raton: CRC Press, 661.

[ref13] Gelman A. Hennig C. (2017). Beyond subjective and objective in statistics. J. R. Stat. Soc. Ser. A Stat. Soc. 180, 967–1033. doi: 10.1111/rssa.12276

[ref14] Gelman A. Jakulin A. Pittau M. G. Su Y.-S. (2008). A weakly informative default prior distribution for logistic and other regression models. Ann. Appl. Stat. 2, 1360–1383. doi: 10.1214/08-AOAS191

[ref15] Ghosh J. Li Y. Mitra R. (2018). On the use of Cauchy prior distributions for Bayesian logistic regression. Bayesian Anal. 13, 359–383. doi: 10.1214/17-BA1051

[ref16] Goodrich B. Gabry J. Ali I. Brilleman S. Rstanarm: Bayesian applied regression modeling via Stan (2024). Available online at: https://mc-stan.org/rstanarm/ (Accessed June 6, 2026).

[ref17] Heck D. W. Boehm U. Böing-Messing F. Bürkner P.-C. Derks K. Dienes Z. . (2023). A review of applications of the Bayes factor in psychological research. Psychol. Methods 28, 558–579. doi: 10.1037/met0000454, 35298215

[ref18] Ibrahim J. G. Chen M. Gwon Y. Chen F. (2015). The power prior: theory and applications. Stat. Med. 34, 3724–3749. doi: 10.1002/sim.6728, 26346180 PMC4626399

[ref19] Jones D. E. Trangucci R. N. Chen Y. (2022). Quantifying observed prior impact. Bayesian Anal. 17, 737–764. doi: 10.1214/21-ba1271

[ref20] Kallioinen N. Paananen T. Bürkner P.-C. Vehtari A. (2024). Detecting and diagnosing prior and likelihood sensitivity with power-scaling. Stat. Comput. 34:57. doi: 10.1007/s11222-023-10366-5

[ref21] Kosmidis I. Firth D. (2021). Jeffreys-prior penalty, finiteness and shrinkage in binomial-response generalized linear models. Biometrika 108, 71–82. doi: 10.1093/biomet/asaa052

[ref22] Kröpelin T. F. Neyens J. C. L. Halfens R. J. G. Kempen G. I. J. M. Hamers J. P. H. (2013). Fall determinants in older long-term care residents with dementia: a systematic review. Int. Psychogeriatr. 25, 549–563. doi: 10.1017/s1041610212001937, 23253253

[ref23] Kruschke J. K. (2015). Doing Bayesian data Analysis: a Tutorial with R, JAGS, and Stan. 2nd Edn Amsterdam: Elsevier, Academic Press, 759.

[ref24] Kruschke J. K. (2021). Bayesian analysis reporting guidelines. Nat. Hum. Behav. 5, 1282–1291. doi: 10.1038/s41562-021-01177-7, 34400814 PMC8526359

[ref25] Lemoine N. P. (2019). Moving beyond noninformative priors: why and how to choose weakly informative priors in Bayesian analyses. Oikos 128, 912–928. doi: 10.1111/oik.05985

[ref26] Lüdecke D. Ben-Shachar M. Patil I. Waggoner P. Makowski D. (2021). Performance: an R package for assessment, comparison and testing of statistical models. J. Open Source Softw. 6:3139. doi: 10.21105/joss.03139

[ref27] Lüdecke D. Kofahl C. (2020). Einsatz von sedierenden Medikamenten und bewegungseinschränkenden Maßnahmen bei Patienten mit Demenz im Akutkrankenhaus: Eine nichtrandomisierte Fall-Kontroll-Studie. Z Gerontol Geriat 53, 138–144. doi: 10.1007/s00391-020-01697-3, 32048012 PMC8279997

[ref28] Lüdecke D. Makowski D. (2025). Choosing informative priors in Bayesian regression models. A simulation study and tutorial using Stan and R. Code and data. doi: 10.17605/OSF.IO/8EVY5 PMC13341808 42421736

[ref29] Lüdecke D. Poppele G. Klein J. Kofahl C. (2019). Quality of life of patients with dementia in acute hospitals in Germany: a non-randomised, case–control study comparing a regular ward with a special care ward with dementia care concept. BMJ Open 9:e030743. doi: 10.1136/bmjopen-2019-030743, 31494617 PMC6731932

[ref30] Mahoney F. I. Barthel D. W. (1965). Functional evaluation: the Barthel index. Md. State Med. J. 14, 61–65. 14258950

[ref31] Makowski D. Ben-Shachar M. S. Chen S. H. A. Lüdecke D. (2019). Indices of effect existence and significance in the Bayesian framework. Front. Psychol. 10:2767. doi: 10.3389/fpsyg.2019.02767, 31920819 PMC6914840

[ref32] Makowski D. Ben-Shachar M. Lüdecke D. (2019). bayestestR: describing effects and their uncertainty, existence and significance within the Bayesian framework. J. Open Source Softw. 4:1541. doi: 10.21105/joss.01541

[ref33] Makowski D. Ben-Shachar M. S. Wiernik B. M. Patil I. Thériault R. Lüdecke D. (2025). Modelbased: an R package to make the most out of your statistical models through marginal means, marginal effects, and model predictions. JOSS 10:7969. doi: 10.21105/joss.07969

[ref34] Mansournia M. A. Geroldinger A. Greenland S. Heinze G. (2018). Separation in logistic regression: causes, consequences, and control. Am. J. Epidemiol. 187, 864–870. doi: 10.1093/aje/kwx299, 29020135

[ref35] McElreath R. (2016). Statistical Rethinking: a Bayesian course with Examples in R and Stan. Boca Raton London New York: CRC Press, Taylor & Francis Group, 469.

[ref36] McNeish D. (2016). On using Bayesian methods to address small sample problems. Struct. Equ. Model. 23, 750–773. doi: 10.1080/10705511.2016.1186549

[ref37] McNeish D. (2019). Two-level dynamic structural equation models with small samples. Struct. Equ. Model. Multidiscip. J. 26, 948–966. doi: 10.1080/10705511.2019.1578657, 32863699 PMC7451754

[ref38] Monachan D. Vargese S. S. Johny V. Mathew E. (2020). Risk of fall among older adults and its association with cognitive impairment in a semi-Urban Community. Indian J. Community Med. 45, 463–466. doi: 10.4103/ijcm.IJCM_491_19, 33623202 PMC7877416

[ref39] Morey R. D. Rouder J. N. (2011). Bayes factor approaches for testing interval null hypotheses. Psychol. Methods 16, 406–419. doi: 10.1037/a0024377, 21787084

[ref40] Muehlemann N. Zhou T. Mukherjee R. Hossain M. I. Roychoudhury S. Russek-Cohen E. (2023). A tutorial on modern Bayesian methods in clinical trials. Ther. Innov. Regul. Sci. 57, 402–416. doi: 10.1007/s43441-023-00515-3, 37081374 PMC10117244

[ref41] Nikolakopoulos S. (2016). Hybrid Bayesian - Frequentist Approaches for Randomized trial design in small Populations. [Doctoral thesis 1 (Research UU / Graduation UU)]. Utrecht, Netherlands: UMC Utrecht.

[ref42] Petersen J. D. Siersma V. D. Christensen R. d P. Storsveen M. M. Nielsen C. T. Waldorff F. B. (2018). The risk of fall accidents for home dwellers with dementia—a register- and population-based case-control study. Alzheimers Dement. Diagn. Assess. Dis. Monit. 10, 421–428. doi: 10.1016/j.dadm.2018.05.004, 30151421 PMC6107894

[ref43] R Core Team (2025). R: A Language and Environment for Statistical Computing. Vienna, Austria: R Foundation for Statistical Computing.

[ref44] Robert C. P. (2007). The Bayesian Choice: From Decision-Theoretic Foundations to Computational Implementation. New York, NY: Springer New York.

[ref45] Rouder J. N. Lu J. (2005). An introduction to Bayesian hierarchical models with an application in the theory of signal detection. Psychon. Bull. Rev. 12, 573–604. doi: 10.3758/BF03196750, 16447374

[ref46] Siddique J. Aghabazaz Z. (2023). Prior ground: selection of prior distributions when analyzing clinical trial data using Bayesian methods. NEJM Evidence 2:EVIDe2300250. doi: 10.1056/EVIDe2300250, 38320533 PMC11197078

[ref47] Šinkovec H. Heinze G. Blagus R. Geroldinger A. (2021). To tune or not to tune, a case study of ridge logistic regression in small or sparse datasets. BMC Med. Res. Methodol. 21:199. doi: 10.1186/s12874-021-01374-y, 34592945 PMC8482588

[ref48] Stan Development Team Stan reference manual, v2.39 (2026). Available online at: https://mc-stan.org (Accessed June 8, 2026)

[ref49] Stefan A. M. Katsimpokis D. Gronau Q. F. Wagenmakers E.-J. (2022). Expert agreement in prior elicitation and its effects on Bayesian inference. Psychon. Bull. Rev. 29, 1776–1794. doi: 10.3758/s13423-022-02074-4, 35378671 PMC9568464

[ref50] Tängman S. Eriksson S. Gustafson Y. Lundin-Olsson L. (2010). Precipitating factors for falls among patients with dementia on a psychogeriatric ward. Int. Psychogeriatr. 22, 641–649. doi: 10.1017/s1041610209991724, 20122302

[ref51] Thirard R. Ascione R. Blazeby J. M. Rogers C. A. (2020). Integrating expert opinions with clinical trial data to analyse low-powered subgroup analyses: a Bayesian analysis of the VeRDiCT trial. BMC Med. Res. Methodol. 20:300. doi: 10.1186/s12874-020-01178-6, 33302878 PMC7727208

[ref52] Tibshirani R. (1996). Regression shrinkage and selection via the lasso. J. Royal Statistical Society Series B: Statistical Methodol. 58, 267–288.

[ref53] Van De Schoot R. Broere J. J. Perryck K. H. Zondervan-Zwijnenburg M. Van Loey N. E. (2015). Analyzing small data sets using Bayesian estimation: the case of posttraumatic stress symptoms following mechanical ventilation in burn survivors. Eur. J. Psychotraumatol. 6. doi: 10.3402/ejpt.v6.25216, 25765534 PMC4357639

[ref54] van de Schoot R. Depaoli S. King R. Kramer B. Märtens K. Tadesse M. G. . (2021). Bayesian statistics and modelling. Nat. Rev. Methods Primers 1:1. doi: 10.1038/s43586-020-00001-2

[ref55] van Doorn C. Gruber-Baldini A. L. Zimmerman S. Hebel J. R. Port C. L. Baumgarten M. . (2003). Dementia as a risk factor for falls and fall injuries among nursing home residents. J. Am. Geriatr. Soc. 51, 1213–1218. doi: 10.1046/j.1532-5415.2003.51404.x, 12919232

[ref56] Van Erp S. Oberski D. L. Mulder J. (2019). Shrinkage priors for Bayesian penalized regression. J. Math. Psychol. 89, 31–50. doi: 10.1016/j.jmp.2018.12.004

[ref57] Vehtari A. Gelman A. Simpson D. Carpenter B. Bürkner P.-C. (2021). Rank-normalization, folding, and localization: an improved Rˆ for assessing convergence of MCMC (with discussion). Bayesian Anal. 16, 667–718. doi: 10.1214/20-BA1221

[ref58] Wickham H. (2016). ggplot2: Elegant Graphics for Data Analysis. 2nd Edn New York: Springer, 260.

[ref59] Zampieri F. G. Casey J. D. Shankar-Hari M. Harrell F. E. Harhay M. O. (2021). Using Bayesian methods to augment the interpretation of critical care trials. An overview of theory and example reanalysis of the alveolar recruitment for acute respiratory distress syndrome trial. Am. J. Respir. Crit. Care Med. 203, 543–552. doi: 10.1164/rccm.202006-2381CP, 33270526 PMC7924582

[ref60] Zondervan-Zwijnenburg M. Peeters M. Depaoli S. Van De Schoot R. (2017). Where do priors come from? Applying guidelines to construct informative priors in small sample research. Res. Hum. Dev. 14, 305–320. doi: 10.1080/15427609.2017.1370966

