# Supplementary material for: Choosing informative priors in Bayesian regression models: a simulation study and tutorial using Stan and R
Source: Front Psychol. 2026 Jun 24;17:1856582. doi: 10.3389/fpsyg.2026.1856582 (PMC13341808; doi:10.3389/fpsyg.2026.1856582)
Supplement: Supplementary file 1 [file Table_1.DOCX]

Supplementary Material

**Supplementary Table 1:** Glossary of important terms in Bayesian modelling used in this paper

| **Term** | **Description** |
| --- | --- |
| Parameter | A number that defines a model's characteristics. For instance, regression coefficients (e.g., β) are parameters that show the strength and direction of a relationship between variables. Models also have other parameters, like sigma (σ), which measures the model's error or spread. |
| Effect size | A statistical measure that quantifies the strength of a relationship between variables, in regression modelling typically between a predictor and an outcome |
| Prior distribution | A probability distribution that represents one's initial belief or existing knowledge about an unknown parameter before considering the current data. The prior is specified as a distribution (not a single number) to express uncertainty about this belief. |
| Location / Scale | These are properties, or “hyperparameters”, that define a (prior) distribution. For instance, a normal distribution is characterized by its mean (location) and standard deviation (scale). When we incorporate prior knowledge, we make assumptions about the most likely value for the parameter (the location) and about the range of other plausible values (the scale). |
| Likelihood function | The likelihood function quantifies how plausible the observed data are, given a specific potential value for a parameter. It is the component of Bayes’ theorem that represents the evidence or information from the data. For example, it answers the question: “If the true odds ratio (our parameter) were 3.0, what is the probability of observing the data we collected?” |
| Posterior distribution | The posterior distribution represents our updated belief about a model parameter after we have considered the evidence (the data). It is the result of combining our initial belief (prior distribution) with what we learned from our data (likelihood).  The posterior distribution is a collection of thousands of parameter estimates generated by a computational sampling process. This distribution represents all plausible values for a parameter based on the model. We can summarize this distribution to make inferences: the mean or median provides the single best estimate (like a regression coefficient), while quantiles create an uncertainty interval showing a range of credible values.  Using summary statistics like mean or quantiles is also common in frequentist regression modelling, e.g. after bootstrapping, which produces “bootstrap samples” for our parameter values. |

*Definitions are adapted from standard Bayesian textbooks, including Gelman et al. (2014) and Kruschke (2015).*

Gelman A, Carlin JB, Stern HS, Dunson DB, Vehtari A, Rubin DB. Bayesian data analysis. Third edition. Boca Raton: CRC Press; 2014.

Kruschke JK. Doing Bayesian data analysis: a tutorial with R, JAGS, and Stan. 2. ed. Amsterdam: Elsevier, Academic Press; 2015.

***Supplementary Figure 1:*** *Posterior median and 95% credible intervals by sample size, prior location and prior scale for true effect of 0.3. Results from 100 regression models with simulated data for each combination of sample size, location, and scale. Narrower prior scales strongly pull estimates toward the prior location at smaller sample sizes. As sample size increases, the data dominates the prior, allowing the estimates to converge on the true effect regardless of the initial prior location. Credible intervals clearly demonstrate the low coverage of the true effect for pessimistic priors in small sample sizes.*


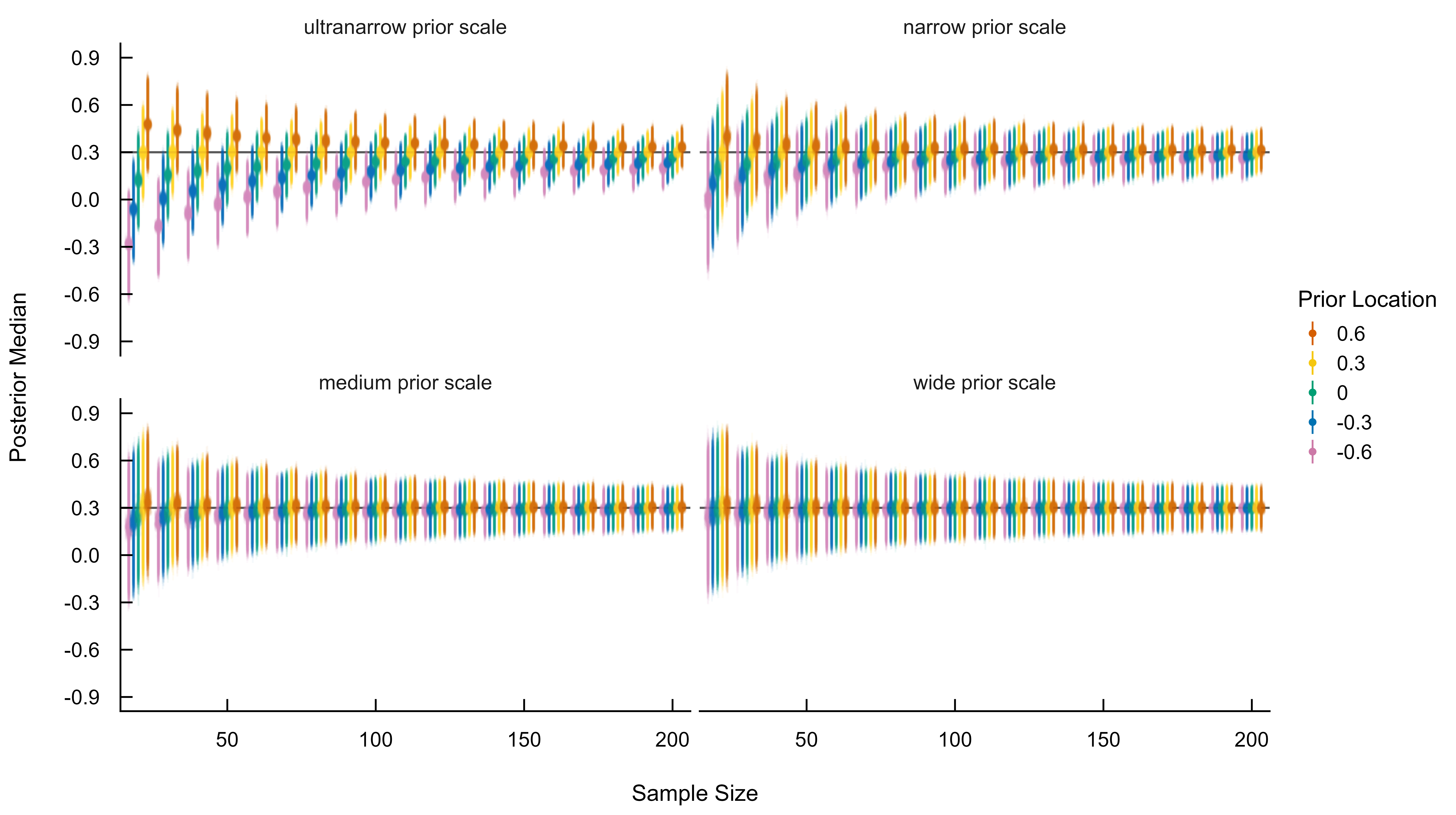


***Working code example in R***

library(easystats)

library(ggplot2)

library(rstanarm)

# Data preparation ----------------------------------------------

# download data from https://doi.org/10.17605/OSF.IO/8EVY5

d <- data_read("Example2/Study 2 - Dataset.rds")

# re-scale quantitative predictors so we can set a global prior on all of the

# nuisance predictors

d <- standardize(d, select = c("stay", "age", "cci_c", "barthel_code"))

# Prior-Assumptions ----------------------------------------------

## for the intercept ------------

# - Fall Incidence in hospitals for reference category (no/mild dementia) ~ 5%

prob_fall <- qlogis(0.05)

prob_fall_scale <- 0.5

prior_intercept <- student_t(

df = 5,

location = prob_fall,

scale = prob_fall_scale

)

# scale = 0.5 (on linear scale) allows a variation of about an assumed range of

# fall incidents from ~ 2% to 13%:

plogis(prob_fall + prob_fall_scale * qt(c(0.05, 0.95), df = 5))

## for the odds-ratios ---------

# moderate dementia means 2fold, strong dementia 3,5fold higher odds of falling

# (OR of 2 resp. 3.5 from other research findings - on the log-scale we get

# approx. the location-values of .7 and 1.25)

prob_dem_mid <- log(2)

prob_dem_hi <- log(3.5)

set.seed(1207)

mf1 <- formula(

fall_incidence ~ stay +

age +

mmse +

cci_c +

barthel_code +

fall_risk +

cam_score +

sex +

group +

chemicalres

)

# Model ------------------------

## believer priors on dementia -----------------------------

exp(prob_dem_hi + 0.5 * qt(c(0.05, 0.95), df = 5))

m_believer <- stan_glm(

mf1,

data = d,

family = binomial("logit"),

prior = student_t(

df = 5,

location = c(0, 0, prob_dem_mid, prob_dem_hi, 0, 0, 0, 0, 0, 0, 0),

scale = c(1, 1, 0.5, 0.5, 1, 1, 1, 1, 1, 1, 1)

),

prior_intercept = prior_intercept,

# don't show model-fitting progress in console

refresh = 0,

open_progress = FALSE,

cores = 4,

seed = 1207

)

# Prior predictive checks ------------------------

# prior predictive checks work by simply estimating the relation between

# the predictor of interest (here: mmse) and the outcome (here: fall incidence)

# based on the prior predictive distribution. There are many functions to do

# this, e.g. using `rstanarm::posterior_epred()`. `estimate_relation()` from

# the modelbased package is a convenient wrapper around these functions, which

# works for dozens of modeling packages.

#

# For Bayesian models, it is important to keep the draws (sampels) from the

# prior predictive distribution for visualization. This is done by setting

# keep_iterations = TRUE.

## believer priors on dementia (same as above) -----------------

# We need to set `prior_PD = TRUE` for the model fitting, to only

# sample from the prior predictive distribution.

prob_dem_mid <- log(2)

prob_dem_hi <- log(3.5)

m_believer_ppc <- stan_glm(

mf1,

data = d,

family = binomial("logit"),

prior = student_t(

df = 5,

location = c(0, 0, prob_dem_mid, prob_dem_hi, 0, 0, 0, 0, 0, 0, 0),

scale = c(1, 1, 0.5, 0.5, 1, 1, 1, 1, 1, 1, 1)

),

prior_intercept = prior_intercept,

# only sample from the prior predictive distribution

prior_PD = TRUE,

# don't show model-fitting progress in console

refresh = 0,

open_progress = FALSE,

cores = 4,

seed = 1207

)

## Predictions from prior distribution -------------------

out_believer <- modelbased::estimate_relation(

m_believer_ppc,

by = "mmse",

keep_iterations = TRUE

)

# reshape iterations for plotting. These are by default in wide format, but

# for ggplot, we need them in long format.

d_believer <- reshape_iterations(out_believer)

# we convert mmse to a factor with other labels that are used for the plot axes

d_believer$mmse <- factor(

as.numeric(d_believer$mmse),

labels = c("mild", "moderate", "severe")

)

## plot function ---------------------

# size and alpha of plot elements

dot_size <- 0.95

dot_alpha <- 0.15

# base plot

ggplot(

d_believer,

aes(x = mmse, group = iter_group, y = iter_value, color = mmse, fill = mmse)

) +

# dots - geom_jitter2 from the see package avoids overplotting and has

# some nicer dots

geom_jitter2(alpha = dot_alpha, size = dot_size) +

# overlay with boxplots, to emphasize probability mass

geom_boxplot(

aes(group = NULL),

alpha = 0.35,

fill = "white",

outliers = FALSE,

width = 0.4

) +

# no legend needed, axis labels angled for better readability

theme_modern(legend.position = "none", axis.text.angle = 45) +

# y axis in percent format

scale_y_continuous(labels = scales::percent_format()) +

# custom colors for the different levels of mmse. we use the color-blind

# friendly okabeito palette from the see package

scale_color_manual(

values = c(

mild = unname(see::okabeito_colors("green")),

moderate = unname(see::okabeito_colors("blue")),

severe = unname(see::okabeito_colors("red"))

)

) +

scale_fill_manual(

values = c(

mild = unname(see::okabeito_colors("green")),

moderate = unname(see::okabeito_colors("blue")),

severe = unname(see::okabeito_colors("red"))

)

) +

labs(x = NULL, y = NULL, title = "Believer")
